# The Relationship Between Gynecological Examination Anxiety, Genital Self‐Image, and Depression, Anxiety, and Stress of Women

**DOI:** 10.1155/da/9021768

**Published:** 2026-08-21

**Authors:** Hilal Gül Boyraz Yanık, Özlem Akın Yamak, Nülüfer Erbil

**Affiliations:** ^1^ Department of Obstetrics and Gynecologic Nursing, Faculty of Health Sciences, Ordu University, Ordu, Türkiye, odu.edu.tr; ^2^ Department of Obstetrics and Gynecologic Nursing, Faculty of Health Sciences, Recep Tayyip Erdoğan University, Rize, Türkiye, erdogan.edu.tr

**Keywords:** anxiety, depression, genital self-image, gynecological examination, stress

## Abstract

**Background:**

Gynecological examination anxiety can be influenced by women’s mental state and genital self‐image. This study aimed to examine the relationship between gynecological examination anxiety, genital self‐image, and depression, anxiety, and stress in women.

**Methods:**

This descriptive and correlational study included 281 women reached through social media. Data were collected using a personal information form, the female genital self‐image scale (FGSIS), the gynecological examination anxiety scale, and the Depression Anxiety Stress Scale‐21.

**Results:**

The mean age of the women was 42.18 ± 11.57. It was found that 63% of the women were university graduates, 29.5% had chronic diseases, 13.5% had gynecological diseases, 59.1% did not have regular gynecological examinations, and 46.3% had undergone a gynecological examination in the last year. The prevalence rates for severe and very severe depression, anxiety, and stress were 7.5%, 15.7%, and 6.4%, respectively. This study found a low‐level negative correlation between genital self‐image and gynecological examination anxiety (*r* = −0.141, *p*  < 0.05). According to the multiple linear regression analysis, previous negative experiences during gynecological examinations were associated with higher anxiety (*β* = 0.129, *p* = 0.033), whereas higher female genital self‐image was associated with lower anxiety (*β* = −0.140, *p* = 0.027).

**Conclusion:**

Previous negative experiences with gynecological examinations can increase gynecological examination anxiety, while a more positive genital self‐image can play a protective role in reducing anxiety.

## 1. Introduction

Gynecological examination is a physical examination of a woman’s external and internal genital organs by a healthcare professional [1, 2], and unlike other examination methods, it affects women both physically and psychologically [3]. Although the examination duration is shorter than that of other medical assessments, it can elicit negative emotions in many individuals and is often perceived as an inappropriate procedure [4, 5]. Women generally express a loss of control during this process and, especially in emergency examinations, experience feelings such as anxiety, shame, fear, pain, embarrassment, and concerns about cleanliness and personal hygiene regarding the detection of diseases or pathological conditions [6, 7]. The use of instruments such as speculums, lack of information about the examination method, and discomfort experienced during the examination of genital organs—which individuals believe should be covered, hidden, and protected—can further complicate the process [8–10]. Therefore, gynecological examinations may be perceived as harmful, leading to reactions such as avoidance of examinations and reluctance to seek routine and diagnostic healthcare services [8, 10].

Many factors influence women’s decisions to undergo regular gynecological examinations. Studies have reported that women’s reasons for avoiding gynecological examinations include feelings of loss of control over their bodies, the examiner being of the opposite sex, the presence of multiple individuals in the examination room, negative perceptions of their genitalia, concerns about genital hygiene and odor, and the fear of experiencing pain during diagnosis and examination [11–15]. Increased concerns about violations of personal privacy and a greater focus on privacy issues can lead to increased anxiety levels and stress and negatively impact mental well‐being in women [16].

Anxiety is a significant factor that can prevent women from making the best use of healthcare services [17]. Gynecological examinations, while a routine part of a woman’s life, are often described as a medical procedure that frequently causes anxiety [18]. Studies in the literature report that women generally experience moderate anxiety before gynecological examinations [5, 9, 16, 17, 19–21]. Furthermore, a systematic review of 15 studies indicated that between 10% and 80% of women experienced fear, embarrassment, and anxiety related to gynecological examinations [22]. Research, particularly concerning genital self‐image, shows that some women avoid or postpone regular gynecological examinations due to anxiety about healthcare professionals visually evaluating their genital area [23].

A significant number of women report feelings of shame, anxiety about their health, fear of pain, and discomfort related to the exposure of their bodies. These emotional responses can negatively affect women’s willingness to participate in gynecological examinations and seek care. One study indicated that women’s genital self‐perception is a factor affecting participation in examinations [24]. In the study conducted by Timur Taşhan et al. [10], 24% of women stated that they had their first gynecological examination, 73.6% stated that they felt embarrassed during the examination, 61.6% stated that they experienced stress, and 74.8% stated that they felt uncomfortable due to the exposure of their private parts. In another study, it was determined that more than half of the women expressed anxiety about their health during pelvic examinations, ~42% experienced feelings of shame, and 18% were afraid of coming to the examination in their clothes and experiencing pain during the procedure [25]. Fathnezhad‐Kazemi et al. [19] revealed that 74.1% of women reported feelings of shame and embarrassment during gynecological examinations, and ~60% experienced stress. This process appears to be not only a physical but also a significantly psychological experience, suggesting that feelings of shame and stress can negatively impact women’s body image and genital self‐image.

Genital self‐image, considered a subdimension of body image, is a subjective concept encompassing an individual’s feelings, attitudes, and experiences regarding their genital organs [26, 27]. Genital self‐perception, on the other hand, includes a person’s thoughts and feelings about the appearance and function of their genital area and constitutes a fundamental component of the genital self‐image [28]. At the societal level, the pressure to conform to idealized and flawlessly presented vulvar and vaginal appearances may lead women to perceive their completely normal and biologically diverse genital anatomy as inadequate [29, 30]. Conversely, it has been reported that a positive genital self‐image increases the willingness of health professionals to evaluate the genital area [26]. Women who are dissatisfied with their genital appearance tend to postpone or not schedule clinical appointments at all, which can reduce their access to preventive health services [28].

Accordingly, women may experience anxiety about participating in gynecological examinations, and the identification of potential problems may be delayed, leading to negative outcomes. Women’s anxiety about gynecological examinations and their genital self‐image may interact with their mood levels; these psychological states can increase the severity of anxiety, intensifying avoidance behaviors and feelings of discomfort related to the examination.

In the current literature, gynecological examination anxiety has generally been addressed as a unidimensional phenomenon, and its relationship with sociodemographic data has mostly been examined [25, 31]. However, studies examining the relationship between gynecological examination anxiety and genital self‐image, a significant component of women’s body perception, are also limited [24]. Furthermore, the role of psychological variables such as depression, anxiety, and stress in this process has been evaluated separately in most studies, and holistic approaches that consider these variables together have not been sufficiently investigated [32, 33]. In this context, it can be said that there is a need for more research into studies that address gynecological examination anxiety, together with its psychological and body image dimensions. This study aims to examine the relationship between women’s gynecological examination anxiety levels, genital self‐image, depression, anxiety, and stress.

### 1.1. Research Questions

• What is women’s genital self‐image like?
• What is the level of anxiety that women experience during gynecological examinations?
• What are the levels of depression, anxiety, and stress among women?
• Is there a relationship between women’s anxiety levels during gynecological examinations and their levels of depression, anxiety, and stress?
• Which factors are associated with women’s anxiety during gynecological examinations?

## 2. Method

### 2.1. Type of Study

The study was planned as a descriptive and correlational study.

### 2.2. Population and Sample of the Study

The study’s sample population consisted of married women from across Türkiye. The sample size was calculated using the unknown population sampling formula. When calculating the sample size, the prevalence of moderate anxiety during gynecological examination was determined as 20.7% (21%), referencing the study by Aksu and Turgut [14]. Using the unknown population sampling method, the number of women to be included in the study was found to be 255. Considering the possibility of exclusion and data loss during data collection, a sample of 281 women, 10% larger than the calculated number, was selected.

### 2.3. Inclusion Criteria

Women aged 18 years and older who were literate, married, and able to use the internet and a smartphone were included.

### 2.4. Exclusion Criteria

Women with a diagnosed mental illness and those who agreed to participate in the study but wished to withdraw at any stage were excluded.

### 2.5. Data Collection

Study data were collected online via a questionnaire form using WhatsApp and social media, based on online self‐reporting, between January 10, 2026, and February 6, 2026. Collecting each data point took ~15 min. Questions were activated after women clicked the consent button before starting the survey.

### 2.6. Data Collection Tools

#### 2.6.1. Personal Information Form

The form, developed by the researchers based on the literature, consists of questions designed to determine women’s sociodemographic and gynecological characteristics, including age, employment status, chronic disease status, diagnosis of a gynecological disease, and experience with gynecological surgery [14, 34, 35].

#### 2.6.2. The Female Genital Self‐Image Scale (FGSIS)

The FGSIS was developed in the United States by Herbenick and Reece [36] to determine women’s perceptions of their genital self‐image. FGSIS consists of 7 items. The Likert‐type scale used a scale of 1–4, where 1 represents “Strongly disagree” and 4 represents “Strongly agree.” The possible score range was 7–28. A higher total score on the FGSIS indicates a positive genital self‐image. Cronbach’s alpha coefficient in the original study of the scale was 0.88 [36]. The validity and reliability of the scale in Turkish were established by Karadeniz and Yangın [37], and the Cronbach’s alpha coefficient of the scale was stated as 0.90 in the original study [37]. In this study, Cronbach’s alpha coefficient was found to be 0.78.

#### 2.6.3. Gynecological Examination Anxiety Scale

The scale was developed by Demirtop et al. [38] to determine the gynecological examination anxiety levels of sexually active women. The scale consists of 20 questions and five subdimensions (health personnel’s approach, health personnel’s experience, negative experiences, hygienic reasons, and individual attitudes). The scale, which has a five‐point Likert structure, does not contain any reverse items. The scale is scored as follows: “Strongly agree = 5, agree = 4, undecided = 3, disagree = 2, and strongly disagree = 1,” with a minimum score of 20 and a maximum score of 100. Higher scores are interpreted as indicating a high level of anxiety. The Cronbach’s alpha value of the scale was found to be *α* = 0.86 [38]. In this study, Cronbach’s alpha value was found to be 0.78.

#### 2.6.4. Depression Anxiety Stress Scale‐21

The Depression Anxiety Stress Scale‐21 was developed by Lovibond and Lovibond [39], and its Turkish validity and reliability study were conducted by Sarıçam [40]. The scale consists of 21 items, with 7 items in each of the depression, anxiety, and stress subscales. The scale evaluates the symptoms of depression, anxiety, and stress in the last week on a four‐point scale from (0) never to (3) always. A score of 5 or higher on the depression subscale, 4 or higher on the anxiety subscale, and 8 or higher on the stress subscale indicates that the individual has a relevant problem. (0–4) points indicate normal, (5–6) mild, (7–10) moderate, (11–13) severe, and 14 and above indicate very severe depression. (0–3) indicates normal, (4–5) mild, (6–7) moderate, (8–9) severe, and 10 and above indicate very severe anxiety. (0–7) indicates normal, (8–9) mild, (10–12) moderate, (13–16) severe, and 17 and above indicate very severe stress [40]. In this study, Cronbach’s alpha internal consistency reliability coefficients were found to be 0.85 for depression, 0.76 for anxiety, and 0.78 for stress [40].

### 2.7. Data Analysis

Data analysis was performed using IBM SPSS Statistics for Windows, Version 29.0 [41]. The normality of the data distribution was evaluated using the Kolmogorov–Smirnov test. Descriptive statistical methods, including frequency, percentage, mean, standard deviation, minimum, and maximum values, as well as parametric tests, were used in the evaluation of the data. Differences in independent groups were evaluated using parametric tests such as the *t*‐test and the one‐way ANOVA test. The relationship between continuous variables was evaluated using Pearson correlation analysis in parametric data. Multiple linear regression analyses were conducted to examine factors associated with gynecological examination anxiety. Categorical variables were transformed into dummy variables (0 = reference category and 1 = comparison category) and entered into the model together with continuous variables. Cronbach’s alpha coefficients were used for the reliability analyses of the scales. A *p*  < 0.05 value was taken as the threshold for statistical significance.

### 2.8. Ethical Aspects of the Research

Permission was obtained via email from the authors who developed the scales used as data collection tools. Ethical approval was obtained from the Ordu University Non‐Interventional Health Sciences Research Ethics Committee for the research (decision date: 29.12.2025 and decision number: 2025/55). The principles of the Helsinki Declaration were adhered to in the research. Women who volunteered to participate in the research were asked to give their consent on the consent form in the online questionnaire.

## 3. Results

The average age of the women included in the study was 42.18 ± 11.57 (range: 22–75). 63.0% of the women were university graduates, 59.4% had a spouse with a university degree, and 54.4% were employed. 70.5% of the women did not have any chronic disease, and 86.5% did not have a diagnosed gynecological disease (Table 1). It was found that 70.1% of the women had no experience with gynecological surgery, 59.1% did not have regular gynecological examinations, and 46.3% had their last gynecological examination within the last year. It was found that 67.6% had not experienced any negative or uncomfortable incident during a gynecological examination before, and 52.0% did not have a preferred physician for gynecological examinations (Table 2). University graduates had higher average FGSIS scores than high school graduates, and employed women had higher average FGSIS scores than unemployed women, and the differences were statistically significant (*p*  < 0.05) (Table 1). Women in the study who regularly underwent gynecological examinations, those who had a gynecological examination in the last year, and those who preferred a male gynecological examiner had higher average FGSIS scores (*p*  < 0.05) (Table 2). There were no differences in average FGSIS scores according to other sociodemographic and gynecological characteristics of women (*p*  > 0.05).

**Table 1:** Distribution of average scores according to some characteristics of women.

Characteristics	*n*	Percentage	GEAS mean ± SD	FGSIS mean ± SD	DASS‐21 depression mean ± SD	DASS‐21 anxiety mean ± SD	DASS‐21 stress mean ± SD
Age							
18–27^a^	18	6.5	68.94 ± 10.97	19.77 ± 4.02	5.77 ± 4.08	5.11 ± 2.72	7.55 ± 3.88
28–37^b^	97	34.5	66.10 ± 11.96	18.81 ± 3.53	5.93 ± 4.21	5.14 ± 3.36	7.58 ± 3.53
38–47^c^	76	27.0	65.57 ± 10.59	18.94 ± 2.90	4.63 ± 3.23	4.31 ± 3.13	5.81 ± 3.55
48 years and older^d^	90	32.0	67.62 ± 13.22	18.43 ± 3.76	5.55 ± 3.35	4.70 ± 2.61	6.65 ± 2.67
Test and *p*	—	—	*F* = 0.687, *p* = 0.561	*F* = 0.847, *p* = 0.469	*F* = 1.880, *p* = 0.133	*F* = 1.146, *p* = 0.331	***F* = 4.443**, **p** = 0.005 **between b and c**
Educational status							
Primary school^a^	44	15.6	68.40 ± 15.60	17.93 ± 3.55	5.79 ± 3.17	5.77 ± 2.72	7.00 ± 3.08
High school^b^	60	21.4	66.53 ± 11.38	17.85 ± 3.63	6.65 ± 4.79	5.75 ± 3.54	7.48 ± 3.82
University^c^	177	63.0	66.22 ± 11.12	19.32 ± 3.32	4.96 ± 3.31	4.19 ± 2.79	6.53 ± 3.26
Test and *p*	—	—	*F* = 0.591, *p* = 0.55	***F* = 5.762**, **p** = 0.004 **between b and c**	***F* = 5.008**, **p** = 0.007 **between b and c**	***F* = 9.130**, **p** < 0.001 **between a and c** **between b and c**	*F* = 1.882, *p* = 0.154
Spouse’s educational status							
Primary school^a^	44	15.7	65.18 ± 12.31	17.50 ± 4.28	6.11 ± 3.62	5.31 ± 2.58	6.97 ± 3.18
High school^b^	70	24.9	67.24 ± 12.68	18.87 ± 3.24	6.20 ± 4.08	5.14 ± 3.71	7.15 ± 3.68
University^c^	167	59.4	66.75 ± 11.59	19.02 ± 3.32	4.96 ± 3.50	4.47 ± 2.81	6.61 ± 3.29
Test and *p*	—	—	*F* = 0.422, *p* = 0.66	*F* = 2.395, *p* = 0.093	***F* = 3.632**, **p** = 0.028 **between b and c**	*F* = 2.017, *p* = 0.135	*F* = 0.697, *p* = 0.449
Employment status							
Employed	153	54.4	66.47 ± 11.23	19.31 ± 3.45	5.02 ± 3.72	4.47 ± 3.07	6.50 ± 3.56
Unemployed	128	45.6	66.81 ± 12.82	18.16 ± 3.43	5.96 ± 3.64	5.14 ± 2.97	7.16 ± 3.10
Test and *p*	—	—	*t* = ‐0.234, *p* = 0.815	***t* = 2.785**, **p** = 0.006	***t* = 0.708**, **p** = 0.035	*t* = 0.863, *p* = 0.392	*t* = −1.624, *p* = 0.101
Chronic illness							
Yes	83	29.5	68.37 ± 11.99	18.49 ± 3.80	6.34 ± 3.90	5.46 ± 3.28	7.25 ± 3.49
No	198	70.5	65.89 ± 11.90	18.91 ± 3.34	5.07 ± 3.57	4.48 ± 2.89	6.62 ± 3.31
Test and *p*	—	—	*t* = 1.586, *p* = 0.11	*t* = ‐0.921, *p* = 0.358	***t* = 2.653**, **p** = 0.012	***t* = 2.499**, **p** = 0.019	*t* = 1.435, *p* = 0.163
Gynecological disease							
Yes	38	13.5	68.15 ± 9.78	17.89 ± 3.63	6.47 ± 4.26	5.63 ± 2.99	7.36 ± 3.98
No	243	86.5	66.39 ± 12.26	18.93 ± 3.44	5.29 ± 3.59	4.64 ± 3.03	6.72 ± 3.26
Test and *p*	—	—	*t* = 0.846, *p* = 0.398	*t* = −1.708, *p* = 0.089	*t* = 1.833, *p* = 0.112	*t* = 1.873, *p* = 0.06	*t* = 1.102, *p* = 0.271

*Note:* Statistically significant values are shown in bold. Alphabetical superscripts (a,b,c,d) are used to identify groups. *F* = ANOVA test; *t* = *t*‐test.

Abbreviation: SD, standard deviation.

**Table 2:** Distribution of average scores according to some characteristics of women (continued).

Characteristics	*n*	Percentage	GEAS mean ± SD	FGSIS mean ± SD	DASS‐21 depression mean ± SD	DASS‐21 anxiety mean ± SD	DASS‐21 stress mean ± SD
Experience with gynecological surgery							
Yes	84	29.9	68.71 ± 11.73	18.53 ± 3.07	6.28 ± 4.11	5.41 ± 3.24	7.25 ± 3.55
No	197	70.1	65.74 ± 11.97	18.89 ± 3.65	5.09 ± 3.47	4.50 ± 2.91	6.61 ± 3.28
Test and *p*	—	—	*t* = 1.916, *p* = 0.056	*t* = ‐0.798, *p* = 0.426	***t* = 2.482**, **p** = 0.022	***t* = 2.323**, **p** = 0.028	*t* = 1.438, *p* = 0.152
Regular GYN examinations							
Yes	115	40.9	67.28 ± 11.11	19.82 ± 3.29	4.77 ± 3.28	4.32 ± 2.90	6.28 ± 3.32
No	166	59.1	66.17 ± 12.52	18.07 ± 3.44	5.92 ± 3.92	5.09 ± 3.10	7.16 ± 3.36
Test and *p*	—	—	*t* = 0.766, *p* = 0.444	***t* = 4.271**, **p** < 0.001	***t* = −2.575**, **p** = 0.008	***t* = −2.094**, **p** = 0.035	***t* = −2.169**, **p** = 0.031
Last GYN examination							
Within the last year^a^	130	46.3	66.45 ± 10.78	19.34 ± 3.54	4.89 ± 3.35	4.64 ± 3.07	6.40 ± 3.45
Within 1–3 years^b^	68	24.2	66.75 ± 13.18	18.25 ± 3.29	5.91 ± 4.36	4.64 ± 2.96	7.14 ± 3.40
More than 3 years^c^	56	19.9	66.98 ± 12.50	17.91 ± 3.35	6.10 ± 3.50	5.19 ± 3.02	7.21 ± 3.19
No examination has been performed^d^	27	9.6	66.44 ± 13.55	19.29 ± 3.52	5.62 ± 3.77	4.85 ± 3.21	7.03 ± 3.21
Test and *p*	—	—	*F* = 0.030, *p* = 0.993	***F = 3.090***, **p** = 0.028 **between a and c**	*F* = 1.956, *p* = 0.121	*F* = 0.479, *p* = 0.697	*F* = 1.154, *p* = 0.328
Negative GYN experience							
Yes	64	22.8	69.65 ± 11.91	18.32 ± 3.73	6.12 ± 4.70	5.00 ± 3.28	7.12 ± 3.89
No	190	67.6	65.98 ± 11.83	19.10 ± 3.45	5.12 ± 3.23	4.57 ± 2.91	6.66 ± 3.18
I do not remember	27	9.6	64.00 ± 12.05	17.70 ± 2.78	6.14 ± 4.00	5.66 ± 3.29	7.03 ± 3.39
Test and *p*	—	—	*F* = 3.021, *p* = 0.05	*F* = 2.653, *p* = 0.072	*F* = 2.280, *p* = 0.104	*F* = 1.759, *p* = 0.174	*F* = 0.506, *p* = 0.604
GYN physician gender preference							
I prefer a female doctor^a^	128	45.5	67.64 ± 12.86	18.39 ± 3.37	6.03 ± 3.68	5.12 ± 3.32	7.03 ± 3.30
I prefer a male doctor^b^	7	2.5	65.71 ± 7.43	21.42 ± 2.37	3.57 ± 1.71	1.85 ± 1.67	3.14 ± 1.67
I have no preference^c^	146	52.0	65.78 ± 11.25	19.00 ± 3.57	5.03 ± 3.73	4.60 ± 2.74	6.78 ± 3.40
Test and *p*	—	—	*F* = 0.851, *p* = 0.428	***F* = 3.141**, **p** = 0.045 **between a and b**	***F* = 3.443**, **p** = 0.033 **between a and b**	***F* = 4.384**, **p** = 0.013 **between a and b, between b and c**	***F* = 4.527**, **p** = 0.012 **between a and b, between b and c**

*Note:* Statistically significant values are shown in bold. Alphabetical superscripts (a,b,c,d) are used to identify groups. *F* = ANOVA test; *t* = *t*‐test.

Abbreviation: SD, standard deviation.

Statistically significant differences were found in DASS‐21 depression subscale scores among women based on their education status, spouse’s education level, employment status, and having a chronic illness (*p*  < 0.05) (Table 1). Those with a high school education level had higher DASS‐21 depression subscale scores than those with a university education status, those who were unemployed had higher scores than those who were employed, and those with a chronic illness had higher scores than those without a chronic illness, all of which were statistically significant. Those with a primary school education had higher scores than those with a university education, those with a high school education had higher scores than those with a university education, and those with a chronic illness had higher DASS‐21 anxiety subscale scores than those without a chronic illness, all of which were statistically significant (*p*  < 0.05) (Table 1).

Women with experience in gynecological surgery had higher mean scores on the DASS‐21 depression and anxiety subscales compared to those without, those who did not have regular gynecological examinations compared to those who did, and those who preferred female gynecological physicians compared to those who preferred male physicians; these differences were found to be statistically significant (*p*  < 0.05) (Table 2). Women aged 28–37 had higher mean scores on the DASS‐21 stress subscale compared to women aged 38–47; this difference was also statistically significant (*p*  < 0.05) (Table 1).

Furthermore, those who did not have regular gynecological examinations had higher mean DASS‐21 stress subscale scores than those who did, those who preferred female gynecological physicians had higher scores than those who preferred male physicians, and those who preferred male physicians had higher scores than those who had no preference (*p*  < 0.05) (Table 2).

**Figure 1:**
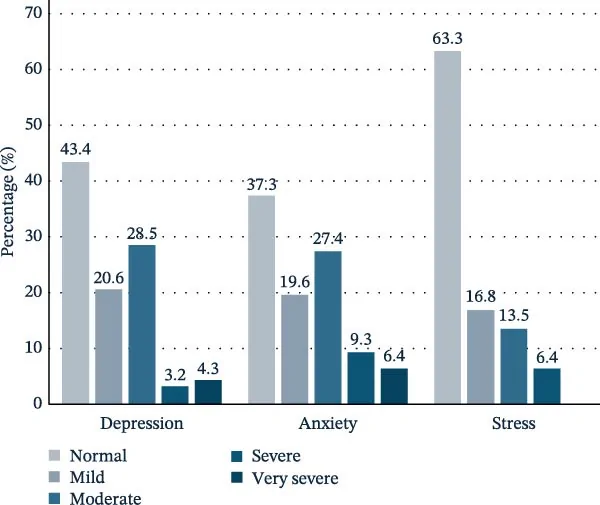
Women’s depression, anxiety, and stress levels (%).

The total score on the gynecological examination anxiety scale was 66.62 ± 11.96 (min: 20–max: 100), indicating above‐moderate anxiety; the total score on the FGSIS was 18.79 ± 3.48 (min: 7–max: 28); and the mean scores on the DASS‐21 depression subscale were 5.45 ± 3.71 (mild depression), 4.77 ± 3.04 (mild anxiety), and 6.80 ± 3.37 (normal level) (Table 3). The prevalence rates for severe and very severe depression, anxiety, and stress were 7.5%, 15.7%, and 6.4%, respectively (Figure 1).

**Table 3:** Mean scores and minimum–maximum values obtained by pregnant women from the scales.

Scales		Min–max scores on the scale	Min–max values obtained	Mean ± SD
Gynecological examination anxiety scale		20–100	40–99	66.62 ± 11.96
GEAS subscales	Approach of health personnel	4–20	4–20	12.51 ± 4.17
	Experience of health personnel	5–25	5–25	19.00 ± 5.89
	Negative experiences	5–25	6–25	15.62 ± 3.91
	Hygienic reasons	3–15	3–15	10.39 ± 3.23
	Individual attitudes	3–15	3–15	9.08 ± 2.54
Female genital self‐image scale		7–28	10–28	18.79 ± 3.48
DASS‐21 subscales	Depression	0–21	0–21	5.45 ± 3.71
	Anxiety	0–21	0–16	4.77 ± 3.04
	Stress	0–21	0–16	6.80 ± 3.37

In this study, a low‐level negative correlation was found between female genital self‐image and gynecological examination anxiety (*r* = −0.141, *p*  < 0.05), and a low‐level negative correlation was found between the DASS‐21 depression subscale and the FGSIS (*r* = −0.320, *p*  < 0.001). A low‐level negative correlation was found between the DASS‐21 anxiety subscale and the FGSIS (*r* = −0.295), and a low‐level negative correlation was found between the DASS‐21 stress subscale and the genital self‐image scale (*r* = −0.246, *p*  < 0.001). A high‐level positive correlation was found between depression and anxiety (*r* = 0.712), a high‐level positive correlation between depression and stress (*r* = 0.730), and a moderate‐level positive correlation between stress and anxiety (*r* = 0.681, *p*  < 0.001), (Table 4)

**Table 4:** Relationship between gynecological examination anxiety, female genital self‐image, and depression, anxiety, and stress.

Scales		Gynecological examination anxiety scale	Female genital self‐image scale	DASS‐depression	DASS‐anxiety	DASS‐stress
Female genital self‐image scale	*r*	−0.141	–	—	—	—
	*p*	0.018	–	—	—	—
DASS‐depression	*r*	0.081	−0.320	–	—	—
	*p*	0.175	<0.001	–	—	—
DASS‐anxiety	*r*	0.083	−0.295	0.712	–	—
	*p*	0.165	<0.001	<0.001	–	—
DASS‐stress	*r*	0.079	−0.246	0.730	0.681	–
	*p*	0.185	<0.001	<0.001	<0.001	–

*Note: r*, Pearson correlation coefficient.

Multiple linear regression analysis revealed that previous negative experiences during gynecological examinations were significantly associated with higher levels of gynecological examination anxiety (*β* = 0.129, *p* = 0.033), whereas higher female genital self‐image was significantly associated with lower anxiety (*β* = −0.140, *p* = 0.027), (Table 5). None of the other variables, including age, educational level, having a chronic illness, diagnosed gynecological disease, experience with gynecological surgery, regular gynecological examination status, time since the last examination, and physician gender preference for gynecological examinations, showed any statistically significant association with anxiety (*p*  > 0.05). In addition, hierarchical regression analysis was conducted to examine the contribution of variable blocks, and the results were largely consistent with the main analysis (see Table S1).

**Table 5:** Multiple linear regression analysis of factors associated with gynecological examination anxiety.

Coefficients^a^						
Model		Unstandardized coefficients		Standardized coefficients	*t*	Sig.
		*B*	Std. error	*β*		
1	Constant	73.363	5.572	—	13.166	<0.001
	Age	0.014	0.068	0.014	0.208	0.836
	Educational status (reference: primary school and high school)	−0.084	1.619	−0.003	−.052	0.959
	Chronic illness (reference: yes)	1.793	1.701	0.069	1.054	0.293
	Gynecological disease (reference: yes)	−1.382	2.348	−0.040	−0.589	0.557
	Experience with gynecological surgery (reference: yes)	2.856	1.696	0.109	1.684	0.093
	Regular GYN examinations (reference: no)	−2.576	1.694	−0.106	−1.520	0.130
	Last GYN examination (reference: more than 3 years and no examination)	1.611	1.779	0.062	0.905	0.366
	Negative GYN experience (reference: yes)	3.663	1.714	0.129	2.137	**0.033**
	GYN physician gender preference (reference: I prefer a female doctor)	1.684	1.454	0.070	1.158	0.248
	Female genital self‐image	−0.482	0.217	−0.140	−2.224	**0.027**

*Note:* Statistically significant values are shown in bold. *R* = 0.257, *R* ^2^ = 0.066, *F* = 1.910, *p* = 0.044.

^a^Dependent variable: GEAS.

## 4. Discussion

Women experience undesirable feelings such as embarrassment and loss of control during gynecological examinations [19]. This study, which examined the relationship between gynecological examination anxiety, genital self‐image, depression, anxiety, and stress, was conducted with 281 women to demonstrate that gynecological examination anxiety is not merely a medical anxiety but consists of a structure related to body and self‐evaluation processes.

The study found that university‐graduate women had a more positive genital self‐image than high school‐graduate women. Although Herbenick et al. [42] found that genital self‐image was not associated with the education level, many other studies have reported similar findings [43–45]. The fact that working women have significantly higher average genital self‐image scores than nonworking women (Table 1) may not be explained by a single factor. This could be related to the group having a higher socioeconomic status, demonstrating stronger psychological resilience, and experiencing lower levels of negative emotions. Furthermore, unemployed women were found to have higher levels of depression compared to employed women. Studies show that depression levels are higher in unemployed women than in employed women [46, 47]. Working women experiencing less emotional distress may develop a more positive body image. Therefore, better emotional health may indirectly contribute to higher genital self‐image scores. Furthermore, the fact that university‐graduate women have a more positive genital self‐image suggests that increased health literacy, body awareness, and critical thinking skills associated with education contribute to a more accepting and positive perception of women’s genital areas. Similarly, the higher genital self‐image scores of working women compared to those of nonworking women suggest that increased social interaction and self‐efficacy perception positively influence genital self‐image.

Genital self‐image is an integral part of body image and reflects an individual’s attitudes, feelings, and related experiences regarding their own genitals [28]. Gynecological examination is described as involving the exposure of the body’s intimate areas in a vulnerable state and with a loss of control [25]. It has been stated that genital self‐image is a factor affecting the frequency of gynecological examinations [24]. In this context, it is an expected finding that those who regularly undergo gynecological examinations and those who have had a gynecological examination in the past year will have higher genital self‐perception scores. A decrease in women’s shame, anxiety, or negative evaluations regarding their genital area may reduce the perception of gynecological examinations as a threatening experience, which could support regular checkups. However, the possibility of a reverse relationship should not be overlooked. In particular, it is hypothesized that in some women with lower genital self‐esteem, frequent gynecological examinations may lead to a habituation process over time, reducing anxiety and shame levels. However, subgroup analyses examining these potential interactions were not conducted in the present study. Therefore, a more detailed investigation of the relationships between gynecological examination frequency, genital self‐esteem, and psychological variables is recommended for future studies.

In addition to these factors, age can also be an important determinant of how gynecological examinations are perceived. Studies support a trend that feelings of shame decrease with increasing age [25, 48]. It was found that 45.5% of the women in this study preferred a female physician for gynecological examinations (Table 2). A study by McLean et al. [49] showed that 96.8% of participants preferred female doctors for gynecological procedures. In the study by Karaküçük and Sönmez [50], it was determined that 56.5% of women preferred female doctors for gynecological examinations, and factors such as having completed primary education, embarrassment, and discomfort were influential in this preference. Women’s discomfort with their genital area may affect their choice of a doctor’s gender. The fact that women in this study who preferred male doctors for gynecological examinations had a higher level of genital self‐image compared to those who preferred female doctors suggests that having a more positive and secure self‐perception regarding the genital area may reduce gender‐based anxiety during examinations. Many studies report that women prefer female physicians for gynecological examinations [25, 51, 52] and that women experience anxiety due to the physician being male during gynecological examinations [25]. Relational dynamics, such as spousal demands, can also play a role in women’s physician preferences. In one study, approximately half of the women stated that their spouses influenced their preference for a female physician, while one‐fifth said they would not change their preference even without their spouses’ influence [50]. Societal cultural perceptions, spousal opinions, and religious factors can also influence choices regarding women’s private lives [50]. Another finding of this study is that women who preferred a female physician during gynecological examinations also showed higher scores for depression, anxiety, and stress. It is thought that this gender discrimination in doctor preference is not limited to cultural factors but may also be related to increased psychological sensitivity, which, in turn, may be associated with privacy, shame, or disease diagnosis.

Gynecological examinations are one of the treatment procedures that increase anxiety in women [8]. It is particularly noted that anxiety and stress are triggered when the sense of privacy is compromised [19]. Women who regularly undergo gynecological examinations have higher depression scores, while those who do not have higher anxiety and stress scores. The presence of an existing health problem or related symptoms indicates that increased sensitivity is more pronounced in this group. However, the fact that women who do not regularly undergo gynecological examinations have increased anxiety and stress levels may suggest that the avoidance behavior towards healthcare is based on anxiety and stress. Women who had gynecological examinations within the last year had higher depression scores than those who had them more than 3 years ago. This suggests that gynecological examinations may have been undertaken mostly due to an existing health problem or the presence of symptoms and that this increased depressive mood may be related to healthcare‐seeking behavior.

Another finding in this study is the weak and negative correlation between women’s genital self‐image and gynecological examination anxiety. This suggests that an increase in genital self‐image may reduce gynecological examination anxiety and that women with a more positive perception of their genital area experience less anxiety during the examination process. In this context, the low level of correlation indicates that gynecological examination anxiety cannot be explained solely by genital self‐image; other variables such as cultural factors, past traumatic experiences, and mental health may also play a role in examination anxiety.

The regression analysis indicated that previous negative experiences during gynecological examinations were associated with higher anxiety levels, whereas a higher genital self‐image was associated with lower anxiety. Age, education level, and other sociodemographic/clinical variables were not significantly associated with anxiety. Thus, gynecological examination anxiety appears to comprise both the experiential and subjective components of body perception. Negative experiences during gynecological or obstetric examinations may even cause women to avoid future gynecological visits [53, 54]. This is because negative experiences may have caused the woman to code the examination environment as threatening, involving a loss of control, or containing a violation of privacy. It can also be said that a woman’s self‐assessment and satisfaction level regarding her genital area influence her perception of gynecological examinations.

### 4.1. Limitations and Strengths of the Study

The findings of this study should be considered in light of certain methodological limitations. First, the collection of data online may have limited the sample to women with internet access or who can use social media; this may have reduced the generalizability of the sample and caused bias. Second, the collection of data through self‐report may also bring about social desirability or recall bias. Third, as the study included only married women, the findings may not be generalizable to single, divorced, or women in other types of relationships. Fourth, considering cultural characteristics, the results may not be directly generalizable to different sociocultural samples. Another limitation is that women’s anxiety about gynecological examinations was assessed solely on a scale basis, and qualitative data regarding the causes of anxiety were not collected. It is recommended that future studies include data that comprehensively evaluate participants’ perceptions and experiences regarding gynecological examination anxiety. Furthermore, the conversion of some categorical variables into binary categories may have led to a loss of information. Additionally, the limited explanatory power of the regression model suggests a multidimensional nature of gynecological examination anxiety. And finally, the fact that variables that may affect the results, such as the past negative gynecological experiences of the women included in the study, social and cultural values, and family structure characteristics, were not addressed in detail within the scope of the research, may lead to different interpretations of the findings. Despite these limitations, the study contributes to the literature by addressing the relationships between genital self‐image, psychological symptoms, and gynecological examination anxiety. It is recommended that the findings be supported by multicenter, longitudinal studies covering different sociodemographic groups in the future.

### 4.2. Implications for Clinical Practice

The findings of this study demonstrate that gynecological examination anxiety is related to genital self‐image and psychological symptoms. In clinical practice, it is important to evaluate women not only physically but also psychosocially. It should be considered that the risk of anxiety may be higher in women with a negative genital self‐image, high levels of depression and anxiety, or a history of negative examination experiences.

Empathic communication, adequate information, respect for privacy, and woman‐centered and trauma‐sensitive care approaches that take patient preferences into account can be effective in reducing examination anxiety. Timely referral to mental health services and early identification of at‐risk groups may help promote adherence to regular gynecological examinations.

## 5. Conclusion

This study comprehensively examined the relationships between women’s genital self‐image, psychological symptoms, and gynecological examination anxiety. The findings showed that higher education level and employment status were associated with a more positive genital self‐image. Additionally, lower education level, unemployment, and the presence of chronic diseases were associated with higher depression and anxiety symptoms.

The study results showed that participants who did not have regular gynecological checkups, had experience with gynecological surgery, and preferred female doctors had higher scores for depression, anxiety, and stress, indicating a relationship between gynecological health behaviors and psychological well‐being. A low‐level but significant negative correlation was found between genital self‐image and gynecological examination anxiety. Similarly, significant negative correlations were observed between genital self‐image and depression, anxiety, and stress symptoms. Strong positive correlations were found among the subdimensions of psychological symptoms. In the multiple linear regression analysis, genital self‐image and previous negative gynecological examination experiences were identified as factors associated with gynecological examination anxiety, although the explained variance was limited.

In conclusion, a more negative genital self‐image and previous negative examination experiences appear to play an important role in increased gynecological examination anxiety. The findings suggest that considering women’s body image and previous experiences in gynecological care may contribute to reducing examination anxiety and encouraging regular gynecological checkups. Accordingly, it is recommended that psychosocial assessments be integrated into clinical practice and that woman‐centered, trauma‐informed approaches be adopted.

## Author Contributions

Hilal Gül Boyraz Yanık, Özlem Akın Yamak, and Nülüfer Erbil were responsible for hypothesis development and drafting the manuscript. Hilal Gül Boyraz Yanık, Özlem Akın Yamak, and Nülüfer Erbil played a key role in creating figures and tables. Hilal Gül Boyraz Yanık, Özlem Akın Yamak, and Nülüfer Erbil were responsible for data acquisition and analysis. Hilal Gül Boyraz Yanık, Özlem Akın Yamak, and Nülüfer Erbil were responsible for data interpretation.

## Funding

No funding was received for this study.

## Disclosure

This study was presented as an oral presentation at the 9th International and 20th National Nursing Congress, held in Ankara, Türkiye, from May 14 to 16, 2026. All authors have read and approved the final manuscript.

## Ethics Statement

Permission was obtained via email from the authors who developed the scales used as data collection tools. Ethical approval was obtained from the Ordu University Non‐Interventional Health Sciences Research Ethics Committee for the research (decision date: 29.12.2025 and decision number: 2025/55). The principles of the Helsinki Declaration were adhered to in the research. Women who volunteered to participate in the research were asked to give their consent on the consent form in the online questionnaire.

## Conflicts of Interest

The authors declare no conflicts of interest.

## Supporting Information

Additional supporting information can be found online in the Supporting Information section.

## Supporting information


**Supporting Information** Table S1: Hierarchical multiple linear regression analysis of factors associated with gynecological examination anxiety. According to the results of the hierarchical multiple linear regression analysis, it was determined that the variables included in the final stage of the model significantly explained gynecological examination anxiety (*R* = 0.257, *R* ^2^ = 0.066, adjusted *R* ^2^ = 0.031, *F* = 1.910, and *p* = 0.044). When the third model was examined, it was determined that previous negative experiences during gynecological examinations significantly predicted anxiety levels in a positive direction (*β* = 0.129, *p* = 0.033), while female genital self‐image significantly predicted anxiety in a negative direction (*β* = −0.140, *p* = 0.027). On the other hand, it was determined that age, education level, presence of chronic disease, diagnosed gynecological disease, history of gynecological surgery, regular gynecological examination status, time of last examination, and physician gender preference in gynecological examinations did not have a significant effect on anxiety level (*p* > 0.05).

## Data Availability

The data that support the findings of this study are available upon request from the corresponding author. The data are not publicly available due to privacy or ethical restrictions.

## References

[1] Cappiello J. and Levi A. , The Annual Gynecologic Examination Updated for the 21st Century, Nursing for Women’s Health. (2016) 20, no. 3, 315–319, 10.1016/j.nwh.2016.03.006. 27287359

[2] Ekici H. , Üstün C. , Kara O. F. , Güven D. , and Güven S. E. , Pelvic Examination, Basic Principles in Gynecology, 2020, Academician Bookstore Inc, 1–7.

[3] Sarpkaya D. and Vural G. , The Use of the Way of Knowing Four in Gynaecological Examination in Nursing, Dokuz Eylul University School of Nursing Electronic Journal. (2014) 7, 124–127.

[4] Tancman S. , HaCohen N. , Lazarus G. , Solt I. , and Sagi-Dain L. , Silent voices that must be heard-women’s perceptions of gynecologic examinations, Journal of Psychosomatic Obstetrics & Gynecology. (2022) 43, no. 2, 190–197. 33416005 10.1080/0167482X.2020.1864727

[5] Ulker K. and Kivrak Y. , The Effect of Information About Gynecological Examination on the Anxiety Level of Women Applying to Gynecology Clinics: A Prospective, Randomized, Controlled Study, Iranian Red Crescent Medical Journal. (2016) 18, no. 6, 10.5812/ircmj.23864. PMC5002923 27621913

[6] Ouj U. , Igberase G. O. , Eze J. N. , and Ejikeme B. N. , Perception of Intimate Pelvic Examination by Gynaecological Clinic Attendees in Rural Southeast Nigeria, Archives of Gynecology and Obstetrics. (2011) 284, no. 3, 637–642, 10.1007/s00404-010-1698-4. 20922401

[7] Wijma B. , Gullberg M. , and Kjessler B. , Attitudes Towards Pelvic Examination in a Random Sample of Swedish Women, Acta Obstetricia et Gynecologica Scandinavica. (1998) 77, no. 4, 422–428, 10.1034/j.1600-0412.1998.770411.x. 9598951

[8] O’Laughlin D. J. , Strelow B. , and Fellows N. , et al.Addressing Anxiety and Fear During the Female Pelvic Examination, Journal of Primary Care & Community Health. (2021) 12, 10.1177/2150132721992195, 2150132721992195. PMC7970676 33525968

[9] Erbil N. , Şenkul A. , Sağlam Y. , and Ergül N. , Determination of Attitudes Toward Gynecologic Examination and Anxiety of Turkish Women Before Gynecologic Examination, International Journal of Human Sciences. (2008) 5, no. 1, 1–13.

[10] Timur Tashan S. , Uçar T. , Aksoy Derya Y. , and Kucukkelepce Şimsek D. , Influence of Gynecologic Examination Anxiety on Application Period to Gynecology, Asian Pacific Journal of Health Sciences. (2016) 3, no. 2, 84–91, 10.21276/apjhs.2016.3.2.15.

[11] Özcan H. , Dağlı A. , and Koçak D. Y. , Experiences of Women With Gynecological Examination: The Example of Gümüşhane, Health Care Academician Journal. (2020) 7, no. 3, 188–195.

[12] Tugut N. and Golbasi Z. , Aspects of Emotional and Physical Discomfort in Gynecologic Examination: A Study of Turkish Women, Journal of Obstetrics and Gynaecology Research. (2014) 40, no. 6, 1777–1784, 10.1111/jog.12409. 24888948

[13] Saleh N. , Abu-Gariba M. , Yehoshua I. , and Peleg R. , Barriers to Implementation of a Pelvic Examination Among Family Doctors in Primary Care Clinics, Postgraduate Medicine. (2018) 130, no. 3, 341–347, 10.1080/00325481.2018.1438078. 29405077

[14] Aksu S. and Turgut B. , Kadınların Jinekolojik Muayene Öncesi Anksiyete Düzeyi Ve Etkileyen Faktörler, İnönü Üniversitesi Sağlık Hizmetleri Meslek Yüksek Okulu Dergisi. (2020) 8, no. 3, 688–700, 10.33715/inonusaglik.762143.

[15] Demir S. and Yeşiltepe O. Ü. , Women’s Experiences of Gynecological Examinations and Their Expectations From Healthcare Professionals, Journal of Women’s Health Nursing. (2014) 1, no. 1, 68–79.

[16] Yilmaz F. T. and Demirel G. , The Relationship Between Body Privacy and Anxiety in Women Having Gynecological Examination, Journal of Obstetrics and Gynaecology. (2021) 41, no. 7, 1112–1115, 10.1080/01443615.2020.1835845. 33427553

[17] Altay B. and Kefeli B. , The Effect of Some Variables to the Alleriatation Anxiety of Women Who Came for Gynecologic Examination, Dokuz Eylul University School of Nursing Electronic Journal. (2012) 5, no. 4, 134–141.

[18] Iraola E. , Menard J. P. , and Chariot P. , Experience of Pelvic Examination and Uptake of Gynecological Care Following Domestic or Sexual Violence: A Systematic Review, Trauma, Violence, & Abuse. (2024) 25, no. 5, 4030–4044. 10.1177/15248380241270038 39162217

[19] Fathnezhad-Kazemi A. , Ahmadi-Geshlag M. , and Ranjbar M. , The Link Between the Perception of Body Privacy and Situational Anxiety: A Study of Women’s Attitudes Toward Gynecological Examinations, Journal of Psychosomatic Obstetrics & Gynecology. (2025) 46, no. 1, 10.1080/0167482X.2025.2582409, 2582409. 41241833

[20] Ozbek H. and Sümer H. , The Effects of Midwifery Approach to the Alleviative Anxiety Level of Women Coming Pelvic Examination, Cumhuriyet University Institute of Health Sciences Journal. (2019) 4, 45–54.

[21] Ragab Eid S. , Ragab Abou-Shabana K. , Ahmed Hassan A. , and Elzeblawy Hassan H. , Effect of Pre-Gynecological Examination Counseling Sessions on Relieving Women’s Pain, Discomfort and Enhancing Their Satisfaction, Journal of Nursing Science Benha University. (2023) 4, no. 1, 751–768, 10.21608/jnsbu.2023.279044.

[22] Bloomfield H. E. , Olson A. , and Greer N. , et al.Screening Pelvic Examinations in Asymptomatic, Average-Risk Adult Women: An Evidence Report for a Clinical Practice Guideline From the American College of Physicians, Annals of Internal Medicine. (2014) 161, no. 1, 46–53, 10.7326/M13-2881. 24979449

[23] Stewart E. G. and Spencer P. , The V Book: A Doctor’s Guide to Complete Vulvovaginal Health, 2002, Bantam Books.

[24] DeMaria A. L. , Hollub A. V. , and Herbenick D. , Using Genital Self-Image, Body Image, and Sexual Behaviors to Predict Gynecological Exam Behaviors of College Women, The Journal of Sexual Medicine. (2011) 8, no. 9, 2484–2492, 10.1111/j.1743-6109.2011.02379.x. 21718451

[25] Yanikkerem E. , Özdemir M. , Bingol H. , Tatar A. , and Karadeniz G. , Women’s Attitudes and Expectations Regarding Gynaecological Examination, Midwifery. (2009) 25, no. 5, 500–508, 10.1016/j.midw.2007.08.006. 18086509 PMC2801597

[26] DeMaria A. L. , Hollub A. V. , and Herbenick D. , The Female Genital Self–Image Scale (FGSIS): Validation Among a Sample of Female College Students, The Journal of Sexual Medicine. (2012) 9, no. 3, 708–718, 10.1111/j.1743-6109.2011.02620.x. 22240088

[27] Sharp G. and Tiggemann M. , Educating Women About Normal Female Genital Appearance Variation, Body Image. (2016) 16, 70–78, 10.1016/j.bodyim.2015.11.006. 26723015

[28] DeMaria A. L. , Meier S. J. , and Dykstra C. , It’s Not Perfect But It’s Mine": Genital Self-Image Among Women Living in Italy, Body Image. (2019) 29, 140–148, 10.1016/j.bodyim.2019.03.011. 30959326

[29] Barbara G. , Facchin F. , Meschia M. , and Vercellini P. , The First Cut is the Deepest”: A Psychological, Sexological and Gynecological Perspective on Female Genital Cosmetic Surgery, Acta Obstetricia et Gynecologica Scandinavica. (2015) 94, no. 9, 915–920, 10.1111/aogs.12660. 25891185

[30] Fahs B. , Genital Panics: Constructing the Vagina in Women’s Qualitative Narratives About Pubic Hair, Menstrual Sex, and Vaginal Self–Image, Body Image. (2014) 11, no. 3, 210–218, 10.1016/j.bodyim.2014.03.002. 24958655

[31] Hassan H. E. , Eid S. R. , Hassan A. , and Abou-Shabana K. R. , Study Women’s Knowledge, Pain, Discomfort, and Satisfaction During First Gynecological Examination, The American Journal of the Medical Sciences. (2022) 1, no. 1, 23–33.

[32] Tancman S. , HaCohen N. , Lazarus G. , Solt I. , and Sagi-Dain L. , Silent Voices That Must Be Heard–Women’s Perceptions of Gynecologic Examinations, Journal of Psychosomatic Obstetrics & Gynecology. (2022) 43, no. 2, 190–197, 10.1080/0167482X.2020.1864727. 33416005

[33] Tugut N. , Demirel G. , Baser M. , Ata E. E. , and Karakus S. , Effects of Lavender Scent on Patients’ Anxiety and Pain Levels During Gynecological Examination, Complementary Therapies in Clinical Practice. (2017) 28, 65–69, 10.1016/j.ctcp.2017.05.006. 28779939

[34] Keramat A. , Malary M. , Moosazadeh M. , Bagherian N. , and Rajabi-Shakib M.-R. , Factors Influencing Stress, Anxiety, and Depression Among Iranian Pregnant Women: The Role of Sexual Distress and Genital Self-Image, BMC Pregnancy and Childbirth. (2021) 21, no. 1, 10.1186/s12884-021-03575-1, 87. 33499805 PMC7836496

[35] Sönmez T. , Koç Ö. , and Alver E. , The Effect of Genital Self-Image on Sexual Satisfaction and Stress in Women After Vaginal Delivery, Revista da Associação Médica Brasileira. (2024) 70, no. 10, 10.1590/1806-9282.20240692. PMC11460638 39383395

[36] Herbenick D. and Reece M. , Outcomes Assessment: Development and Validation of the Female Genital Self- Image Scale, The Journal of Sexual Medicine. (2010) 7, no. 5, 1822–1830, 10.1111/j.1743-6109.2010.01728.x. 20233278

[37] Karadeniz Z. C. and Yangın H. , Female Genital Self-Image Scale: Turkish Adaptation and Examination of Its Psychometric Properties, Ordu Üniversitesi Hemşirelik Çalışmaları Dergisi. (2026) 9, no. 2, 198–206, 10.38108/ouhcd.1622865.

[38] Demirtop H. , Beydağ K. D. , and Karaahmet A. Y. , Development and Validation of the Gynecologic Examination Anxiety Scale (GEAS): A 20-Item Patient-Reported Measure, International Journal of Gynecology & Obstetrics. (2026) 1–9, 10.1002/ijgo.71014. PMC13629570 42007601

[39] Lovibond P. F. and Lovibond S. H. , The Structure of Negative Emotional States: Comparison of the Depression Anxiety Stress Scales (DASS) With the Beck Depression and Anxiety Inventories, Behaviour Research and Therapy. (1995) 33, no. 3, 335–343, 10.1016/0005-7967(94)00075-U. 7726811

[40] Sarıçam H. , The Psychometric Properties of Turkish Version of Depression Anxiety Stress Scale-21 (DASS-21) in Health Control and Clinical Samples, Journal of Cognitive-Behavioral Psychotherapy and Research. (2018) 7, no. 1, 19–30, 10.5455/JCBPR.274847.

[41] IBM Corp. , IBM SPSS Statistics for Windows (Version 29.0), 2023, IBM Corp.

[42] Herbenick D. , Schick V. , Reece M. , Sanders S. , Dodge B. , and Fortenberry J. D. , The Female Genital Self-Image Scale (FGSIS): Results From a Nationally Representative Probability Sample of Women in the United States, The Journal of Sexual Medicine. (2011) 8, no. 1, 158–166, 10.1111/j.1743-6109.2010.02071.x. 21044269

[43] Berman L. and Windecker M. A. , The Relationship Between Women’s Genital Self-Image and Female Sexual Function: A National Survey, Current Sexual Health Reports. (2008) 5, no. 4, 199–207, 10.1007/s11930-008-0035-4.

[44] Çataloluk A. and Eroğlu V. , The Relationship Between Genital Self-Image and the Quality of Sexual Life in Married Turkish Women, Sexuality & Culture. (2025) 29, no. 1, 191–209, 10.1007/s12119-024-10277-1.

[45] Sabbaghan M. , Mirghafourvand M. , Hakimi S. , and Malakouti J. , The Relationship Between Socio-Demographic Characteristics With Genital Self-Image in the Women, Journal of Research Development in Nursing and Midwifery. (2021) 18, no. 2, 1400–1404, 10.52547/jgbfnm.18.2.49.

[46] Kurian D. , Depression Among Working Andnon-Workingmiddle Aged Women, Rajagiri Journal of Social Development. (2012) 4, no. 2, 30–34.

[47] Zeab F. and Ali U. , Depression in Working and Non-Working Women in Pakistan: A Comparative Study, Journal of Social Sciences and Humanities. (2018) 57, no. 1, 113–126, 10.46568/jssh.v57i1.107.

[48] Hilden M. , Sidenius K. , Langhoff-Roos J. , Wijma B. , and Schei B. , Women’s Experiences of the Gynecologic Examination: Factors Associated With Discomfort, Acta Obstetricia et Gynecologica Scandinavica. (2003) 82, no. 11, 1030–1036, 10.1034/j.1600-0412.2003.00253.x. 14616277

[49] McLean M. , Al Yahyaei F. , Al Mansoori M. , Al Ameri M. , Al Ahbabi S. , and Bernsen R. , Muslim Women’s Physician Preference: Beyond Obstetrics and Gynecology, Health Care For Women International. (2012) 33, no. 9, 849–876, 10.1080/07399332.2011.645963. 22891743

[50] Karaküçük S. and Sönmez M. , Jinekoloji ve Obstetride Hastaların Hekim Cinsiyeti Tercihleri Ve Etkileyen Faktörler, Sağlık ve Sosyal Refah Araştırmaları Dergisi. (2022) 4, no. 1, 12–22.

[51] Subki A. H. , Agabawi A. K. , and Hindi M. M. , et al.How Relevant is Obstetrician and Gynecologist Gender to Women in Saudi Arabia?, International Journal of Women’s Health. (2021) 13, 919–927, 10.2147/IJWH.S284321. PMC8523902 34703321

[52] Spaich S. , Weiss C. , and Sütterlin M. , Altered Patient Perceptions and Preferences Regarding Male and Female Gynecologists: A Comparison Between 1997 and 2018, Archives of Gynecology and Obstetrics. (2019) 300, no. 5, 1331–1341, 10.1007/s00404-019-05315-5. 31583460

[53] Çalım S.İ. , Ulaş S. C. , Sülüden E. , Ataç N. , Göçer Ş. , and Yürekli Z. N. , Akademisyen Kadınların Jinekolojik Muayene Deneyimleri Ve Beklentilerinin Belirlenmesi, Adnan Menderes Üniversitesi Sağlık Bilimleri Fakültesi Dergisi. (2022) 6, no. 1, 125–134, 10.46237/amusbfd.891444.

[54] Edelstam G. , Makdessi L. , Hagmar M. , Moberg C. , Olovsson M. , and Spira J. , Optimised Gynaecological Examination With a New Pelvic Examination Chair, Sexual & Reproductive Healthcare. (2019) 19, 84–87, 10.1016/j.srhc.2019.01.001. 30928140

