# Supplementary material for: The Relationship Between Gynecological Examination Anxiety, Genital Self‐Image, and Depression, Anxiety, and Stress of Women
Source: Depress Anxiety. 2026 Aug 21;2026:9021768. doi: 10.1155/da/9021768 (PMC13498849; doi:10.1155/da/9021768)
Supplement: Supplementary file 1 [file DA-2026-9021768-s001.docx]

**Table S1.** Hierarchical Multiple Linear Regression Analysis of Factors Associated with Gynecological Examination Anxiety

| **Coefficients^a^** | | | | | | | | | |  | | |
| --- | --- | --- | --- | --- | --- | --- | --- | --- | --- | --- | --- | --- |
| Model | | Unstandardized Coefficients | | Standardized Coefficients | | t | Sig. | Collinearity Statistics | | *R* | *R Square* | *Adjusted R Square* |
|  |  | B | Std. Error | Beta | |  |  | Tolerance | VIF |  |  |  |
| 1 | (Constant) | 64.478 | 2.759 |  | 23.369 | | .000 |  |  | .105 | .011 | −,003 |
|  | Age | .031 | .068 | .030 | .459 | | .646 | .823 | 1.215 |  |  |  |
|  | Educational Status | .300 | 1.609 | .012 | .187 | | .852 | .846 | 1.181 |  |  |  |
|  | Chronic illness | 1.975 | 1.706 | .075 | 1.157 | | .248 | .843 | 1.186 |  |  |  |
|  | Gynecological Disease | 1.001 | 2.159 | .029 | .463 | | .643 | .937 | 1.067 |  |  |  |
| 2 | (Constant) | 62.949 | 3.043 |  | 20.688 | | .000 |  |  | .221 | .049 | .017 |
|  | Age | .023 | .068 | .022 | .332 | | .740 | .803 | 1.245 |  |  |  |
|  | Educational Status | .498 | 1.609 | .020 | .309 | | .757 | .829 | 1.207 |  |  |  |
|  | Chronic illness | 1.690 | 1.712 | .065 | .987 | | .325 | .820 | 1.220 |  |  |  |
|  | Gynecological Disease | −.787 | 2.349 | −.023 | −.335 | | .738 | .775 | 1.290 |  |  |  |
|  | Experience with gynecological surgery | 2.849 | 1.708 | .109 | 1.668 | | .097 | .818 | 1.222 |  |  |  |
|  | Regular GYN examinations | −1.621 | 1.651 | −.067 | −.982 | | .327 | .759 | 1.317 |  |  |  |
|  | Last GYN examination | 1.458 | 1.791 | .056 | .814 | | .416 | .750 | 1.334 |  |  |  |
|  | Negative GYN experience | 3.967 | 1.721 | .139 | 2.305 | | **.022** | .961 | 1.041 |  |  |  |
|  | GYN physician gender preference | 2.021 | 1.457 | .084 | 1.387 | | .167 | .951 | 1.052 |  |  |  |
| 3 | (Constant) | 73.363 | 5.572 |  | 13.166 | | .000 |  |  | .257 | .066 | .031 |
|  | Age | .014 | .068 | .014 | .208 | | .836 | .800 | 1.249 |  |  |  |
|  | Educational Status | −.084 | 1.619 | −.003 | −.052 | | .959 | .807 | 1.239 |  |  |  |
|  | Chronic illness | 1.793 | 1.701 | .069 | 1.054 | | .293 | .819 | 1.221 |  |  |  |
|  | Gynecological Disease | −1.382 | 2.348 | −.040 | −.589 | | .557 | .765 | 1.307 |  |  |  |
|  | Experience with gynecological surgery | 2.856 | 1.696 | .109 | 1.684 | | .093 | .818 | 1.222 |  |  |  |
|  | Regular GYN examinations | −2.576 | 1.694 | −.106 | −1.520 | | .130 | .711 | 1.407 |  |  |  |
|  | Last GYN examination | 1.611 | 1.779 | .062 | .905 | | .366 | .749 | 1.336 |  |  |  |
|  | Negative GYN experience | 3.663 | 1.714 | .129 | 2.137 | | **.033** | .955 | 1.047 |  |  |  |
|  | GYN physician gender preference | 1.684 | 1.454 | .070 | 1.158 | | .248 | .940 | 1.063 |  |  |  |
|  | Female Genital Self-Image | −.482 | .217 | −.140 | −2.224 | | **.027** | .868 | 1.152 |  |  |  |

*Statistically significant values are shown in bold.*

*According to the results of the hierarchical multiple linear regression analysis. it was determined that the variables included in the final stage of the model significantly explained gynecological examination anxiety (R = 0.257. R² = 0.066. adjusted R² = 0.031. F = 1.910. p = 0.044). When the third model was examined. it was determined that previous negative experiences during gynecological examinations significantly predicted anxiety levels in a positive direction (β = 0.129. p = 0.033). while female genital self-image significantly predicted anxiety in a negative direction (β = −0.140. p = 0.027). On the other hand. it was determined that age. education level. presence of chronic disease. diagnosed gynecological disease. history of gynecological surgery. regular gynecological examination status. time of last examination. and physician gender preference in gynecological examinations did not have a significant effect on anxiety level (p > 0.05).*
